# Is Bone Tissue Really Affected by Swimming? A Systematic Review

**DOI:** 10.1371/journal.pone.0070119

**Published:** 2013-08-07

**Authors:** Alejandro Gómez-Bruton, Alejandro Gónzalez-Agüero, Alba Gómez-Cabello, José A. Casajús, Germán Vicente-Rodríguez

**Affiliations:** 1 GENUD “Growth, Exercise, NUtrition and Development” Research Group, Universidad de Zaragoza, Zaragoza, Spain; 2 Faculty of Health and Sport Science (FCSD), Department of Physiatry and Nursing, Universidad de Zaragoza, Huesca, Spain; 3 Department of Sport and Exercise Science, Aberystwyth University, Aberystwyth, United Kingdom; Oklahoma State University, United States of America

## Abstract

**Background:**

Swimming, a sport practiced in hypogravity, has sometimes been associated with decreased bone mass.

**Aim:**

This systematic review aims to summarize and update present knowledge about the effects of swimming on bone mass, structure and metabolism in order to ascertain the effects of this sport on bone tissue.

**Methods:**

A literature search was conducted up to April 2013. A total of 64 studies focusing on swimmers bone mass, structure and metabolism met the inclusion criteria and were included in the review.

**Results:**

It has been generally observed that swimmers present lower bone mineral density than athletes who practise high impact sports and similar values when compared to sedentary controls. However, swimmers have a higher bone turnover than controls resulting in a different structure which in turn results in higher resistance to fracture indexes. Nevertheless, swimming may become highly beneficial regarding bone mass in later stages of life.

**Conclusion:**

Swimming does not seem to negatively affect bone mass, although it may not be one of the best sports to be practised in order to increase this parameter, due to the hypogravity and lack of impact characteristic of this sport. Most of the studies included in this review showed similar bone mineral density values in swimmers and sedentary controls. However, swimmers present a higher bone turnover than sedentary controls that may result in a stronger structure and consequently in a stronger bone.

## Introduction

Osteoporosis and related fractures are a considerable health concern worldwide [1] and cause increased morbidity, mortality and costs for society [2]. This disease is characterized by low bone density and microarchitectural deterioration of bone tissue with a consequent increase in bone fragility and susceptibility to fracture [3]. Adolescence is a critical period for bone acquisition [4], and epidemiological studies have suggested that achieving a high peak of bone mass during growth might decrease the risk of suffering osteoporosis and therefore osteoporotic fractures later in life [5], [6]. In addition to genetic predisposition and physiological factors, calcium and vitamin D intake [4] and having an active lifestyle [7] are among the most important factors related to peak bone mass acquisition. Therefore, physical activity and participation in sport during growth periods are crucial for the acquisition of a high peak of bone mass and to prevent future related pathologies.

The osteogenic effect of exercise is mainly produced by the impacts and mechanical loads applied to the bone. The modelling and remodelling bone turnover process adapts the bone to new demands and, as a consequence, bone mineral content (BMC) and density (BMD) are modified [8], [9]. It is possible that structural and trabecular microarquitecture adaptations are also produced [6]. However, not all physical activities have the same effects on bone; a minimum duration and intensity are required [10], [11] in order for this osteogenic stimulus to be produced.

Recent literature reviews have shown that high impact sports seem to be more osteogenic than non impact sports such as swimming or cycling, in children [12], young adults [12] or older adults [13]. However, to date, systematic reviews compare sports, but none have focused specifically on swimming and bone, an area in which a vast amount of research has recently been produced. Nevertheless, results among studies remain disparate. Recent published studies that have not been included in any previous review show surprising results regarding bone structure in this population [14]–[17]. Therefore, a systematic review in this area is needed in order to elucidate the effect of swimming on bone.

Previous studies performed on swimmers use a variety of techniques to evaluate bone mass and bone metabolism. Dual-energy x-ray absorptiometry (DXA), as a ‘gold standard’ method for measuring bone mass, has been used to evaluate BMC and BMD. Peripheral quantitative computed tomography (pQCT) provides data regarding cortical and trabecular bone and, therefore, internal architecture, geometry and current bone strength. Another technique to evaluate bone parameters in swimmers has been quantitative ultrasound (QUS); its use in young populations is gaining rapid support because results are less likely to be affected by bone size and the technique is less expensive and invasive than other radiologic methods.

The heterogeneity of the studies (i.e. use of different devices, comparison groups, age range…) makes comparisons between studies difficult; however, a systematic review and summary of the available literature on bone mass and swimmers may help to detect possible concerns and to define topics for future research. Therefore the aim of this review was to summarize current knowledge on bone characteristics in swimmers.

## Methods

### Data sources and search strategy

This study followed the systematic review methodology proposed in the Preferred Reporting Items for Systematic reviews and Meta-Analyses (PRISMA) statement [18]. A PRISMA checklist is included (Table S1).

Studies were identified by searching within the electronic databases and consultation with experts in the field. This search was applied to Pubmed, Embase and SportDiscus. The search was conducted up to and including 30 April 2013.

The thesaurus of the words: swimmer*, swimming (always the same descriptor for both terms), bone density, bone and bones were researched in each database. An advanced search was then carried out in which the thesaurus we had found (not always specific for each word) for the “bone terms” were combined with the Boolean operator “OR”. This resulted in a number which was subsequently combined with the number obtained from the “swimming/swimmer” thesaurus searched with the Boolean operator “AND”.

Two reviewers independently examined each database to obtain the potential publications. Relevant articles were obtained in full, and assessed against the inclusion and exclusion criteria described below. Inter-reviewer disagreements were resolved by consensus. Arbitration by a third reviewer was used for unresolved disagreements.

### Inclusion criteria

1. Types of study: Cross-sectional, longitudinal, randomized and non-randomized controlled trials studying the effects of swimming training programmes on bone mass with or without coexistent treatments.
2. Types of participants: children, adolescents, adults and elderly populations.
3. Types of intervention: trials comparing the effects of following or not following an exercise training programme and descriptive cross-sectional or longitudinal studies.
4. Types of outcome measured: BMC and BMD of whole body, lumbar spine, arm, hip (femoral neck, trochanter, intertrochanter and Wards triangle subregions), bone architecture (from pQCT), ultrasound parameters (Broadband Ultrasound Attenuation (BUA), Speed of Sound (SOS), stiffness index) and metabolic biomarkers.

### Exclusion criteria

1) Studies in languages other than English or Spanish 2) Unpublished data 3) Studies with animals 4) Studies without a control group that would permit comparison, and 5) Studies focusing exclusively on bone markers not measuring bone with an imaging technique.

### Search summary

**Figure 1:**
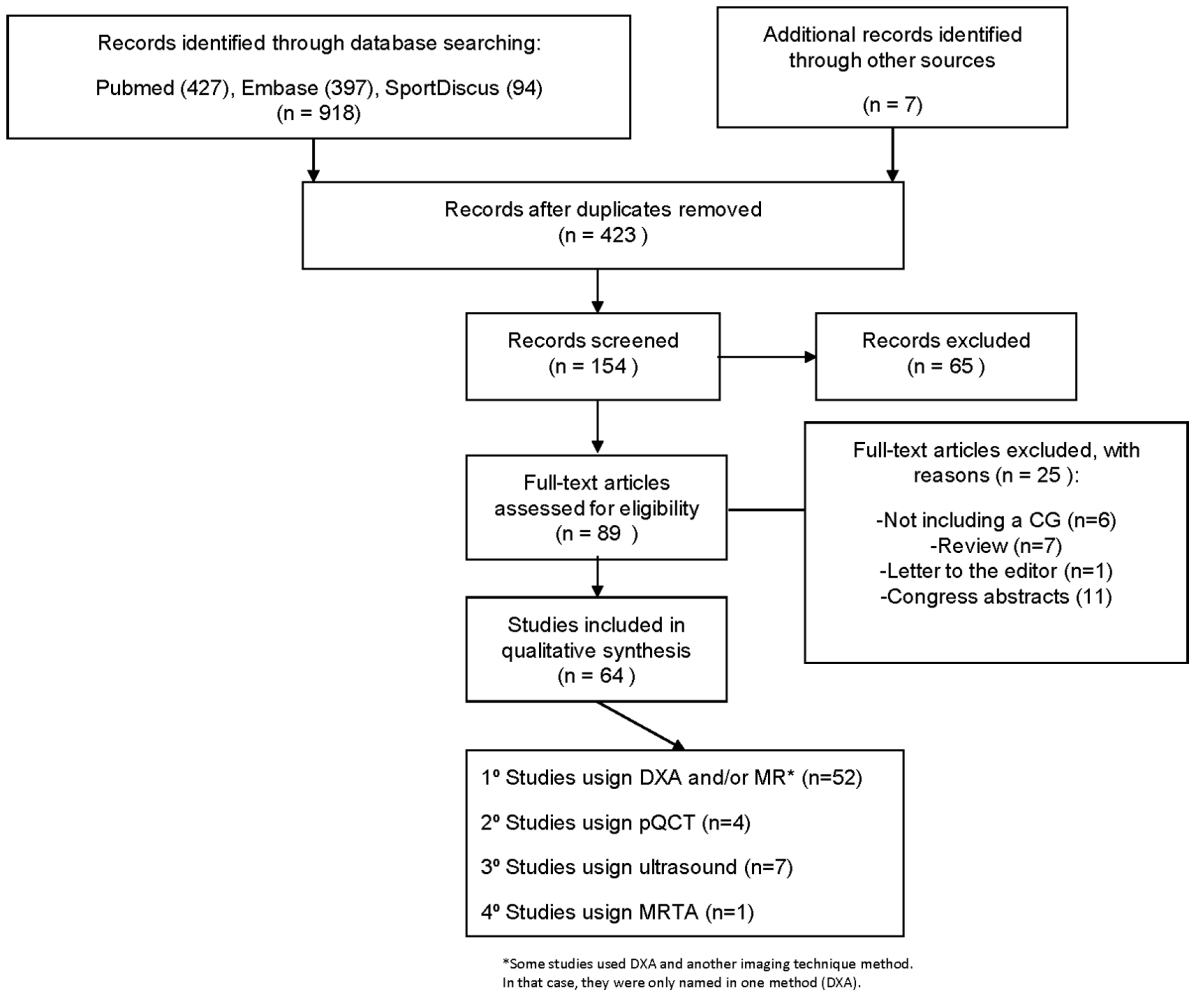
Prisma flow diagram.

Searches identified 423 potentially relevant articles and an additional 7 articles were identified through reference lists. Following review of titles and abstracts and excluding the duplicates, the total was reduced to 154 potentially relevant papers for inclusion. Of these articles, 64 met the selection criteria and were included in this review (Figure 1).

### Quality assessment

Studies were assessed using 2 different quality assessment tools. For cross-sectional studies we used the same quality assessment tool as Olmedillas et al. [19] that grades articles on a scale of 7 points. For longitudinal studies the checklist performed by Tooth et al. [20] was used, classifiying articles on a scale composed of 33 items evaluating the study design and the internal validity.

## Results and Discussion

Results have been divided into two sections; The first section (3.1) organized according to the type of device used for the bone analysis, and the second section (3.2) organized according to factors affecting bone mass. Studies included in this review are summarized in Tables 1–4.

**Table 1:** Studies using photon absorptiometry and magnetic resonance images.

Study	Participants			Study design	Training years	Training hours/week	Data source	Measured areas	Outcome
	Number	Sex	Age						
Nilsson et al.[22] 1971	SWI (9)	M	17.9±4.5	Case-control	–	–	Photon-absorption method	Femur	All athletes had significantly higher femoral BMD compared to CG except SWI that did not significantly differ.
	WLI (11)		20.7±8.4						
	THR (4)		23.5±3.0						
	RUN (25)		22.2±7.1						
	SOC (15)		24.9±5.2						
	CG (39)		22.5±5.1						
Jacobson et al. [42] 1984	SWI (23)	F	23–75	Case-control	≥3	3 times per week	DXA	LSP	SWI had higher radius and metatarsus BMC than CG SWI had lower lumbar spine
	TEN (11)		23–75				SPA	Metatar. Radius	
	OAT (86)		23–75						
	CG		23–75						
Orwoll et al. [43] 1989	SWI (99)	M-F	60±13	Case-control	≥3	≥3	DXA	Vertebra	Male SWI had higher BMD at radial and vertebral measured sites than CG
	CG (119)		60±12				SPA	Radius	Females showed no differences in BMD among groups
							Calcium intake		No differences in calcium intake among groups
Heinrich et al. [113]1990	SWI (13)	F	21.7±3.0	Case-control	10.5	10	DXA	Femur LSP	SWI showed lower BMC than BOD
	CRU (5)		20.2±1.1		4.4	5	SPA	Radius	No differences in BMC among SWI, RUN and CG
	RRU (11)		30.3±4.8		5.5	4	Calcium intake		No differences in calcium intake among groups
	BOD (11)		25.7±5.2		2.5	10			
	CG (18)		25.2±4.4						
Risser et al. [54]1990	SWI (10)	F	18.4±1.3	Case-control	–	17	DXA	LSP	SWI had lower lumbar spine BMD than all other groups including the CG
	VOL (12)		19.6±1.5			18.5	SPA	Calcaneus	SWI had lower calcaneus BMD than VOL and BAS
	BAS (7)		19.6±1.1			17	Calcium intake		No differences in calcium intake among groups
	CG (13)		19.8±1.4						
McCulloch et al. [32] 1992	SWI (20)	M-F	15.0±1.10	Case-control	-	18	SPA	Calcaneus Radius	SWI had lower calcaneum density than SOC
	SOC (23)		15.3±0.77			10			No differences in calcaneum density between SWI and CG
	CG(25)		14.9±0.56						No differences in distal radius BMC between SWI and SOC
							Calcium intake		SWI presented higher calcium intake than SOC and CG
Xia Qu MA [80] 1992	SWI (6)	M	–	Case-control	–	18	X-ray	Humeri	SWI humeri diameter was lower than DIS
	GYM (8)								SWI showed lower cortex humeri than WLI
	JAB (7)								No differences between SWI and CG
	DIS (9)								
	WLI (11)								
	CG (5)								
Grimston et al. [55] 1993	SWI (17)	M-F	12.6±0.4	Case-control	–	–	DXA	FNECK LSP	SWI had lower BMD at femoral neck
	WBE (17)		13.2±0.4						Male swimmers had lower lumbar spine BMD than WBE
							Calcium intake		No differences in calcium intake among groups
Taaffe et al. [61] 1995	SWI (26)	F	19.2±2.1	Case-control	12.2±2.2	22.3±3.3	DXA	FNECK LSP TROCH WB	SWI showed lower whole body BMAD than GYM
	GYM (19)		19.3±1.2		7.5±2.5	21.1±3.8			SWI showed lower femoral neck BMAD than GYM and CG
	CG (19)		19.2±1.6			<3			
Lee et al. [58] 1995	SWI (7)	F	18.9±1.5	Case-control	–	–	DXA	Femur LSP WB WTRI	No difference in BMD between SWI and CG.
	VOL (11)		19.4±1.3						SWI showed lower BMD than BAS and VOL
	BAS (7)		19.9±1.4						SWI showed lower Wards triangle BMD than BAS
	SOC (9)		19.4±1.4						SWI showed lower femoral neck BMD than SOC
	MOD (17)		20.4±1.0				Calcium intake		No differences in calcium intake among groups
	SED (11)		21.6±1.3						
Fehling et al. [36] 1995	SWI (7)	F	20.1±0.8	Case-control	12.3±3.0	20	DXA	Femur LSP WB WTRI	No differences in BMD between SWI and CG
	VOL (8)		19.5±1.3		8.0±2.8	20			SWI showed lower BMD at lumbar spine, femoral neck, wards triangle, legs, pelvis and whole body than VOL and GYM
	GYM (13)		19.6±1.0		9.8±3.0	20			SWI showed lower arm BMD than GYM
	CG (17)		20.8±1.2			<1			
Cassell et al. [23] 1996	SYS (5)	F	9.0±0.2	Case-control	≥1	4.7	DXA	WB	No differences in BMD between SWI and CG
	SSW (9)		9.0±0.2			4.7			SWI showed lower whole body BMD than GYM
	GYM (14)		8.8±0.2			13.9	Calcium intake		No differences in calcium intake among groups
	CG (17)		8.3±0.2						
Dook et al. [44] 1996	SWI (20)	F	42–50	Retrospective	≥20	–	DXA	WB	SWI showed lower whole body and regional leg BMD than HIG
	HIG (20)								SWI showed higher regional arm BMD than CG
	MED (20)						Calcium intake		No difference in calcium intake among groups
	CG (20)								
Matsumoto et al. [94] 1997	SWI (28)	M-F	M 19.2±0.7 F 19.6±1.0	Case-control	≥4	–	DXA	WB	SWI showed lower BMD than JUD
	LDR (38)		M 19.5±0.4 F 20.4±0.4						No differences in BMD between SWI and LDR
	JUD (30)		M 19.8±0.6 F 19.4±0.7				Bone markers		Male SWI showed higher B-ALP values than male LDR
									PICP levels were not different among groups
									Male SWI had lower pyridinoline values than male JUD
									SWI showed lower deoxypiridinoline levels than JUD
Taffe et al. [60]1997	SWI (11)	F	19.0±1.2	12 month follow-up	Start training (years) 7.0±3.5	20	DXA	FNECK LSP WB	SWI femoral neck and whole body BMD was lower than GYM
	GYM (8)		18.9±1.1		Start training (years) 10.5±2.9	20			SWI gained less bone than GYM during the 12 month period
	CG (11)		20.0±2.0						No differences in gained BMD between SWI and CG
Emslader et al. [105] 1998	SWI (22)	F	20.5±0.32	Case-control	≥3	10 miles per week	DXA	FNECK LSP WB	No differences in BMD among groups.
	RUN (21)		20.3±0.36		≥3	40 miles per week			No differences regarding calcium intake among SWI and RUN
	CG (20)		20.4±0.32				Calcium intake		SWI had higher calcium intake than CG
Courteix et al. [26] 1998	SWI (10)	F	10.5±1.4	Case-control	≥3	–	DXA	FNECK Hip LSP Radius TROCH	No differences in BMD between SWI and CG
	GYM (18)		10.4±1.3						SWI showed lower BMD at lumbar spine, femoral neck, wards triangle and overall radius when compaired to GYM
	CG (13)		10.7±1						
Courteix et al. [25] 1998	SWI (10)	F	10.5±1.4	Case-control	≥3	8–12	DXA	FNECK Hip LSP TROCH Radius	No differences in BMD between SWI and CG
	GYM (18)		10.4±1.3			10-15			SWI showed lower BMD at whole body, lumbar spine, femoral neck, wards triangle and overall radius when compared to GYM
	CG (13)		10.7±1			2	Calcium intake		No differences in calcium intake among groups
Taaffe et al. [65]1999	SWI (11)	M	19.9±1.2	Case-control	Start training (years) 10.7±3.7	24.7±4.2	DXA	Femur LSP WB	No differences in BMD among groups
	CG (11)		19.1±1.6			3.4±1.6			
Courteix et al. [24] 1999	SWI (12)	F	10.6±1.1	Case-control	≥3	8–12	DXA	FNECK Head LSP Radius TROCH WTRI	SWI had lower radius, femoral neck and wards triangle BMD than GYM
	GYM (32)		10.15±1.4		≥3	10–15			SWI had higher head BMD and BMC than GYM
	CG (16)		10.5±1.1			2			No differences in BMD between SWI and CG
							Calcium intake		No difference in calcium intake among groups
Kearny [38] 2000	SWI (8)	F	11.0±1.07	Case-control	≥6 months	5–8	DXA	FNECK LSP TROCH WB WTRI	No differences in BMD between SWI and GYM in whole body, hip and lumbar spine.
	GYM (8)		11.75±0.89			15–24			
Creighton et al. [57] 2001	SWI (7)	F	18–26	Case-control	≥4	10–13	DXA	FNECK LSP TROCH WB WTRI	No differences in BMD between SWI and CG
	BAS (8)					10–13			SWI had lower BMD than BAS & VOL at femoral neck, trochanter and total body.
	VOL (6)					10–13			SWI had lower BMD than SOC & TRA at the trochanter and total body
	SOC (9)					6	Bone markers		SWI showed lower bone formation (OC) than BAS, VOL, SOC & TRA
	TRA (4)					14			NTx was higher although not significant in swimmers than in all the other groups
	CG (7)					<1	Calcium intake		No difference in calcium intake among groups
Taffe et al. [41]2001	SWI (10)	M	25.5±5.6	Case-control	–	12	SPA	CalcaneusLeg	SWI had lower values than JUM for BMC and BMD
	JUM (10)		24.9±3.9			12			No differences in BMC and BMD for SWI and CG
	CG (10)		27.7±7.3						
Lima et al. [47] 2001	ALG (27)	M	14.9±1.6	Case-control	4.8±3.1	16.4±4	DXA	FNECK LSP WB	SWI showed higher total BMD and BMC than the CG
	ILG (18)		15.6±1.7		5.7±2.7	17.8±7.6			SWI showed lower lumbar spine and total BMD than the impact group
	CG (24)		15.2±2.0			2–3	Bone markers		SWI showed higher B-ALP levels than the impact group
									SWI showed higher Dpd levels than the CG
							Calcium intake		No differences in calcium intake among groups
Morel et al. [52] 2001	SWI(34)	M	22±3.6	Retrospective	–	8.7±4.8	DXA	Arms Head Legs LSP WB	SWI showed lower total BMD than all the other groups except for ROW
	RUN(126)		34±8.5			8.1±5.3			SWI showed lower arm BMD than FIG and RUG
	RUG(110)		26±5.9			8.7±5.6			SWI showed lower leg BMD than RUG, FIG, TEAM
	TRI(91)		30±8.1			8.3±4.6			
	MMA(65)		33±8.1			4±5.2			
	SOC(47)		29±7.3			6.7±4.8			
	CYC (47)		32±9.3			8.2±4.7			
	FIG (44)		29±8.7			9.1±4.1			
	WBA (44)		34±9.3			7.9±5.3			
	ROW (30)		27±8.2			22.7±7.1			
	BOD (28)		27±5.1			8.1±6.0			
	OTS (20)		27±7.4			10.7±7.3			
	CLIM(18)		26±3.2			10.8±7.4			
Duncan et al. [30] 2002	SWI (15)	F	16.7±1.3	Case-control	6.1	15±4.8	DXA	Arms FNECK Leg LSP WB	No differences in BMD between SWI and CG
	CYC (15)		16.5±1.4		3.1	15±4.9			SWI showed lower BMD at femoral neck, leg and total body than RUN
	RUN (15)		17.6±1.4		5.0	8.4±1.2			SWI showed lower leg BMD than TRI
	TRI (15)		17.7±1.1		2.5	16.2±4.7			
	CG (15)		16.9±0.9			<2			
Duncan et al. [31] 2002	SWI (10)	F	16.7±1.3	Case-control	6.1	15±4.8	DXA	WB	No differences in vBMD, BMC or bone volumes among groups
	CYC (10)		16.5±1.4		3.1	15±4.9	MRI		SWI had a smaller size-adjusted BSI and CSMI than RUN
	RUN (10)		17.6±1.4		5.0	8.4±1.2			No differences in BSI among SWI, CYC, TRI and CG
	TRI (10)		17.7±1.1		2.5	16.2±4.7			SWI had higher size-adjusted medullary cavity CSA and lower cortical CSA compared with RUN and TRI
	CG (10)		16.9±0.9			<2	Calcium intake		No differences in calcium intake among groups
Maimoun et al. [39] 2003	SWI (13)	M	25.4±6.5	Case-control	12.6±5.6	10.7±3.2	DXA	Femur FNECK LSP Radius TROCH	SWI showed no differences in BMD when compared with the other groups
	CYC (11)		27.4±5.8		9.3±6.8	10.6±3.9	Bone markers		SWI showed higher Testosterone levels than CYC
	TRI (14)		25.7±6.6		9.3±6.8	15.2±4.3			No differences in luteininizing hormone, estrogen, free androgen index, sex hormone-binding globulin, and cortisol among athletes
	CG (10)		27.5±4.3			<2	Calcium intake		SWI had higher calcium intake than CG
Maïmoun et al. [40] 2004	SWI (13)	M	25.4±6.5	Case-control	12.6±5.6	10.7±3.2	DXA	Femur FNECK LSP Radius WB	SWI showed no differences in BMD when compared with the other groups
	CYC (11)		27.4±5.8		9.3±6.8	10.6±3.9	Bone markers		OC and CTX concentrations were higher in swimmers than CG
	TRI (14)		25.7±6.6		6.2±2.2	15.2±4.3			B-ALP concentrations were not different among SWI, TRI and CG being higher in these than in CYC
	CG (10)		27.5±4.3			<2			Serum calcium, phosphate, iPTH and 1.25(OH)_2_ vitamin D were similar in the four groups
							Calcium intake		SWI had higher calcium intake than CG
Bellew et al. [68] 2006	SWI (29)	F	12.0±2.1	Case-control	5.2±2.5	–	SPA	Calcaneus	SWI showed lower BMD than SOC
	SOC (16)		15.1±1.2		4.9±1.8				No differences in BMD between SWI and WLI
	WLI (19)		13.6±1.3		5.1±2.4				SWI showed lower BMD than reference values
Magkos et al. [51] 2007	SSW (9)	M	21.0±2.2	Case-control	–	–	DXA	WB	SWI showed lower aBMD at the legs than RUN
	ESW (7)		19.4±1.9						SWI showed lower leg and total body aBMD than the other groups
	SRU (11)		23.4±3.1						The intensity of exercise had significant main effects on aBMD at nearly all regions examined
	ERU (10)		23.4±3.8						
	CG (15)		22.0±3.3						
Magkos et al. [46] 2007	SWI (26)	M-F	M 20.3±0.6 F 19.8±0.8	Case-control	–	–	DXA	WB	SWI showed lower leg and total BMD than CG
	WPO (43)		M 24.6±0.8 F 22.0±0.7						Female SWI had higher arm BMC than CG
	CG (30)		M 22.0±0.9 F 22.9±0.6						Male SWI showed lower leg BMC than CG
Mudd et al. [53] 2007	SWI (9)	F	20.4±1.1	Case-control	–	–	DXA	Leg LSP Pelvis WB	SWI showed lower average leg BMD scores than all other athletes except for RUN and CRW
	GYM (8)		19.7±0.9						SWI showed lower whole body BMD than TRA, SOF, GYM and FHO
	SOF (14)		20.1±1.1						
	RUN (25)		20.4±1.3						
	TRA (8)		20.1±1.3						
	FHO (10)		19.8±1.2						
	SOC (10)		19.8±0.9						
	CRW (15)		20.5±2.1						
Nichols et al. [114] 2007	SWI & REP (68)	F	15.6±1.3	Case-control	–	–	DXA	FNECK Hip LSP TROCH WB	SWI & REP eumenorrheic athletes showed lower total hip and trochanter BMD than HIL eumenorrheic athletes
	HIL (93)		15.6±1.2						SWI & REP oligo\amenorrheic athletes showed lower spine and trochanter BMD than HIL eumenorrheic athletes.
Derman et al. [28] 2008	SWI (40)	M-F	M 10–17 F 9–16	Case-control	>3	2 h/day	DXA	WB	Male SWI had higher BMD Z-scores than CG
	CG (40)		M 10–16 F 10–16						No differences in BMD between male or female SWI and CG
							Bone markers		No differences in the measured biomarkers among groups
							Calcium intake		SWI showed higher calcium intake than CG
Velez et al. [35] 2008	SWI (43)	M-F	≥65	Case-control	–	–	DXA	FNECK Hip LSP Radius WB	No differences in BMD among SWI and CG
	RUN (44)								SWI showed lower hip intertrochanter BMD than RUN
	CG (87)						Calcium intake		Swimmers showed lower calcium intake than runners and higher than the CG
Jürimäe et al. [27] 2009	SWI (28)	M	10–16	Case-control	≥2	8.4±1.7	DXA	LSP WB	BMD increased through puberty with no differences among groups.
	CG (28)						Bone markers		In SWI Ghrelin was the most important hormonal determinant for total BMD and lumbar BMAD
Carbuhn et al. [50] 2010	SWI (16)	F	17–21	1 year follow-up	–	–	DXA	Arm Leg LSP Pelvis	SWI showed lower BMD than all other athletes at preseason and postseason
	SOF (17)		18–22						SWI increased arm, leg, pelvis, spine and total BMD from the preseason to the postseason period
	BAS (10)		18–21						SWI increased total BMC from the preseason to the postseason period
	VOL (7)		19–20						
	TRA, JUM & SRU (17)		17–23						
Gruodytè et al. [56] 2010	SWI (24)	F	13.7±1.2	Case-control	≥2	9.4±3.2	DXA	FNECK LSP	SWI showed lower femoral neck BMD than RYG
	SPG (49)		14.0±0.9			4.8±1.3			SWI showed lower femoral neck BMC than all other groups except for CCS
	TSR (24)		14.3±1.1			4.8±2.2	Bone markers		No differences in visfatin and leptin concentration levels among groups.
	RYG (23)		14.3±1.0			9.6±4.9			SWI showed lower insulin levels, glucose and insulin resistance index than TSR
	CCS (17)		13.9±0.9			6.3±1.1			In SWI none of the adipocytokines measured were found to be related to bone mineral parameters
	CG (33)		14.2±1.1			90 min. of physical education			
Morgan et al. [59] 2011	SWI (11)	F	20.1±1.7	4–6 Month follow-up	–	10–17	DXA	FNECK Hip LSP TROCH WTRI	No differences in BMD between SWI and CG
	BAS (6)		19.7±0.8			10–13			SWI showed lower total hip BMD than BAS and SOC
	SOC (12)		19.2±1.3			8–13			SWI showed lower BMC for femoral neck, trochanter and total hip than BAS
	CG (4)		19.3±1.9			<1			SWI showed lower BMC for femoral neck and total hip than SOC
									SWI showed no differences regarding BMD over time compared with all the other groups
							Bone markers		SWI showed lower B-ALP levels than BAS and SOC
									No differences were found in bone resorption markers (NTx) among groups
							Calcium intake		No differences in calcium intake among groups
Dias Quiterio et al. [29] 2011	SWI (20)	M	16.4±2.5	Case-control	Starting age 8.7±2.8	19.1±6.2	DXA	Limbs LSP WB	No differences in BMD between SWI and CG
	HIG (34)		15.7±1.6		Starting age 8.0±3.8	12.8±8.7			SWI showed lower leg BMD, BMC and BA than HIG
	CG (26)		15.9±2.8						SWI showed lower lumbar spine BMC than HIG
							Calcium intake		No differences in calcium intake among groups
Silva et al. [33] 2011	SWI (12)	M	13.8±2.5	Case-control	≥3	17.3±1.6	DXA	Femur LSP WB	SWI showed lower proximal femur BMD than SOC and TEN
	TEN (10)		14.1±1.6			16.0±0.8			No differences in BMD among swimmers and CG
	SOC (10)		14.7±0.8			15.1±0.8	Calcium intake		No differences in calcium intake among groups
	CG (14)		13.4±2.0						
Dlugolęcka et al. [34] 2011	SWI (41)	F	11.5±1.0	Case-control	2.4±1.2	12.0±3.2	DXA	LSP	No differences in BMD between SWI and CG
	CG (45)		12.0±0.8				Calcium intake		SWI had higher calcium intake than CG although both groups on average did not exceed 49% of normal sufficient consumption
Ferry et al. [14] 2011	SWI (26)	F	15.9±2	Case-control	≥6	10	DXA	FNECK Hip LSP WB	SWI showed lower BMC and BMD than SOC
	SOC (32)		16.2±0.7		≥7	10	HSA	Hip	SWI showed lower values in parameters reflecting bone strength (CSMI, Z, BR) than SOC
									SWI had HSA Z-scores below the normal values of CG
							Calcium intake		SWI had higher calcium intake than SOC
Greenway et al. [37] 2012	SWI (43)	F	40.4±7.9	Retrospective	>5	≥2	DXA	FNECK LSP Radius Tibia WB	No differences in BMD or BMC among groups
	CG (44)		43.8±7.3				Calcium intake		No differences in calcium intake among groups
Andreoli et al. [45] 2012	SWI (12)	F	58.4±8.8	Retrospective	>20	5.1±2.1	DXA	LSP WB	SWI and RUN showed higher BMD values than GC in most of the measured zones
	RUN (12)		57.8±6.4			4.4±1.0			SWI showed lower leg BMD than RUN
	CG (24)		60.8±6.7			3.0±1.0			
Ferry et al. [15] 2012	SWI (26)	F	15.9±2	8 month follow-up	≥6	10	DXA	FNECK Hip LSP WB	SWI showed lower BMD than SOC at baseline and less changes during the longitudinal period
	SOC (32)		16.2±0.7		≥7	10			SWI decreased their BMD Z-score for whole body and lumbar spine
							HSA		SWI did not increase sub-periosteal width while SOC did.
									SWI increased femoral shaft CSA but this increase was higher in SOC
									SWI showed no changes in CSMI and Z Z-scores at femoral shaft section while SOC improved these parameters
									SWI improved Z-score of BR while SOC did not
							Calcium intake		SWI had higher calcium intake than SOC
Czeczuk et al. [49] 2012	SWI1 (11)	F	52.1±3.3	12 month follow-up	Currently not swimming	Current PA 4.8	DXA	LSP	SWI1 and CG1 showed higher BMC and BMD than SWI2 and CG2
	SWI2 (7)		63.3±4.3			Current PA 6.3			SWI1 BMC and BMD decreased less after a year than CG1
	CG1 (11)		50.7±2.2			Current PA 1.4	Calcium intake		SWI1 and SWI2 had higher calcium intake than CG1 and CG2
	CG2 (7)		60.6±2.3			Current PA 0.6			
Hind et al. [67] 2012	SWI (10)	M	23.2±4.3	Case-control	>3	>5	DXA	Femur	No difference in BMD between SWI and the rest of the groups.
	RUN (31)		27.2±4.4		>3	>5	HSA		SWI showed shorter Hip axis length than RUN and CG
	GYM (14)		22.5±2.0		>3	>5			SWI showed lower CSMI than runners
	CG (22)		26.4±5.4						SWI showed lower Femoral Strength Index than runners
Maïmoun et al. [17] 2013	SWI (20)	F	14.1±1.8	Case-control	Starting age 6.5±1.8	14.5±5.9 20.3±4.2 21.1±4.4 2.5±0.5	DXA	FNECK LSP Pelvis Radius Skull TROCH WB	SWI showed lower aBMD than ARG at all the measured sites except for skull
	ARG (20)		13.8±2.0		Starting age 5.6±1.7				SWI showed lower aBMD than RYG at the femoral region
	RYG (20)		13.8±2.2		Starting age 6.6±1.2		HSA		SWI showed lower CSA and mean cortical thickness and higher buckling ratio than ARG and RYG
	CG (20)		13.7±2.0				Bone markers		SWI showed lower RANKL than ARG, RYG and CG due to a lower value in the postmenarcheal period
									No differences were found in PINP, OC, CTX or OPG among groups
Maimoun et al. [16] 2013	SWI (25)	F	12–18.1	12 month follow-up	>5 years	15.2±4.4	DXA	Femur FNECK LSP Radius TROCH	SWI showed higher arm BMD than CG
	CG (21)					1.8±1.2			No differences were found in BMD variation after a year between groups
							HSA Bone markers		No differences were found in the rest of the measured zones nor in the bone geometry or bone markers among groups.
Czeczelewski et al. [66] 2013	SWI (20)	F	11.6±0.9	36 month follow-up	2.3±1.2	11.9±3.7	DXA	LSP	BMC and BMD increased every year without differences between groups
	CG (20)		12.2±0.8			2.2±2.2			No differences in LSP BMD between groups
							Calcium intake		No differences between groups for Calcium intake
Narra et al. [79] 2013	SWI (18)	F	20.2±2.6	Retrospective	10.0±3.8	17.2±5.6	MRI	FN	No differences between SWI and the rest of the groups
	HIG (19)		21.3±3.2		9.8±3.3	11.8±2.8			
	ODD (19)		23.5±5.1		9.9±4.0	7.8±3.1			
	POW (17)		27.5±6.3		8.0±4.7	9.1±2.7			
	RUN (18)		28.9±5.6		12.4±6.7	10.9±3.4			
	CG (20)		24.1±3.4			2.9±1.5			

aBMD =  Areal bone mineral density; ALG =  Active load group(swimming+waterpolo); Aprox =  Approximately; ARG =  Artistic gymnasts; B-ALP =  Bone specific alkaline phosphatase; BA =  Bone area; BAS =  Basketball; BMC =  Bone mineral content; BMAD =  Bone mineral apparent density; BMD =  Bone mineral density; BOD =  Body builders; BR =  Buckling ratio; BSI =  Bone strength index; CCS =  Cross country skiing; CG =  Control group; CG1 =  Post-menopausal control group for less than 5 years; CG2 =  Post-menopausal control group for more than 5 years; CLIM =  Climbing; CRU =  Collegiate runners; CRW =  Crew; CSA =  crosssectional area; CSMI =  Cross-sectional moment of inertia; CTX =  Type I collagen C-telopeptide; CYC =  Cyclists; DIS =  Discus throwers; DIV =  Divers; Dpd =  Deoxypyridinoline; ERU =  Endurance runners; ESW =  Endurance swimmers; F =  Female; FHO =  Field hockey; FIG =  Fighting; FNECK =  Femoral Neck; GYM =  Gymnasts; HIG =  High impact sports; HIL =  High impact sports (soccer, vollyball); HSA =  Hip structural analysis; ILG =  Impact load group; iPTH =  Intact parathormone; JAV =  Javelin throwers; JUM =  Jumpers; JUD =  Judoists; LDR =  Long distance runners; LSP =  Lumbar Spine; M =  Male; MED =  Medium impact (running and field hockey); Metatar =  Metatarsus; MMA =  Multiple mixed activities; MOD =  Moderate; MRI =  Magnetic resonance imaging; NTx =  Cross-linked N-telopeptides of type I collagen; OAT =  Older athletes: OC =  Osteocalcin; ODD =  Odd impact sports; OPG =  Osteoprotegerin; OTS =  Other practiced sports; PICP =  Procollagen type I C-peptide; PA =  Physical activity; PINP =  Procollagen type 1 N-terminal propeptide; Pyd =  Pyridinoline; POW =  Power lifters; REP =  Repetitive impact sports(running); ROW =  Rowing; RRU =  Recreational runners; RUG =  Rugby; RYG =  Rhytmic gymnastics; RUN =  Runners; SWI =  Swimmers; SWI1 =  Post-menopausal swimmers for less than 5 years ; SWI2 =  Post-menopausal swimmers for more than 5 years; SOC =  Soccer; SED =  Sedentary; SOF =  Softball; SPA =  Single photon absorptiometer; SPG =  Sport games; SRU =  Sprint runners; SSW =  Sprint swimmers; SYS =  Synchronized swimmers; THR =  Throwers; TRA =  Track; TEAM =  Team sports; TEN =  Tennis; TRI =  Triathletes; TROCH =  Trochanter; TSR =  Track sprinters; VOL =  Volleyball; W =  Women; WB =  Whole body; WBA =  Weight bearing activities; WBE =  Weight bearing sports; WLI =  Weight lifters; WPO =  Water polo; WTRI =  Wards triangle; Z =  Section modulus.

**Table 2:** Studies using pQCT.

Study	Participants			Study design	Training years	Training hours/week	Data source	Measured areas	Outcome
	Subjects	Sex	Age						
Liu et al. [75] 2003	SWI (30)	M-F	M 19.5±0.7 F 19.4±1.0	Case- control	Age at start of training M I9.8±1.9 F SWI 7.6±1.9	6.0	pQCT	Midtibia	No differences in vBMD of whole and cortical bone among the three male groups.
	JUM (25)		M 19.8±1.3 F 19.9±1.4		M 12.8±2.1 F 12.7±1.5	5.0			In female SWI whole body and cortical vBMD was lower than CG
	CG (25)		M 20.3±1.6 F 20.2±1.4						In female SWI periosteal area, endocortical area, PMI and SSI, where higher than CG
									CSA and PMI of cortical bone in SWI was smaller than in JUM
Nikander et al. [74] 2006	SWI (27)	F	20.6±2.8	Case- control	10.6±4.3	13.5±4.5	pQCT	Radius Tibia	No differences in distal tibia for BMC, PSM and cortical walls between SWI and CG
	VOL (21)		21.2±3.0		8.6±.3.3	9.9±2.5			No differences at tibial shaft for BMC, CSA, PSM and cortical walls between SWI and CG
	HUR (24)		20.2±2.1		10.4±3.0	9.1±2.4			SWI showed higher distal radius, humeral shaft and CSA of the humeral midshaft than CG
	RAC (23)		23.6±4.5		9.6±3.5	4.6±1.9			SWI had higher humeral PSM than CG
	SOC (18)		21.4±3.0		10.7±3.8	8.6±5.5			SWI showed higher cortical BMD at tibial shaft than VOL
	CG (30)		24.3±3.1			2.9±2.0	Calcium intake		No differences in calcium intake among groups
Shaw et al. [73] 2009	SWI (15)	M	21.9±2.5	Case- control	10.3±2.9	13–15 years 9.1±5.1 16– 19 years 9.5±5.7	pQCT	Humerus Radius Ulna	SWI showed higher resistance to TD,CA,Imin than CG for the dominant humerus
	CRI (16)		22.0±2.5		11.6±2.3	13–15 years 14.9±7.6 16–19 years 20.8±10.7			SWI showed greater TA than CG for the dominant humerus
	CG (20)		21.6±4.7						SWI showed higher resistance to Imax, Im, CA, TD and TA than CG for the non-dominant humerus
									SWI showed higher ACT, TA and CA than CRI for the non-dominant humerus
									SWI showed higher TD, Imax and Imin than the CG for the non-dominant ultna
									SWI showed higher Imax, Imin and TD than CG for the dominant ulna for the non-dominant humerus
Nikander et al. [76] 2010	SWI (45)	F	20.2±2.6	Retro spective	10.0±3.8	17.2±5.6	pQCT	Distal tibia Tibial shaft	No differences between SWI and CG for the distal tibia
	HIG (64)		21.3±3.2		9.8±3.3	11.8±2.8			SWI showed lower BMC and CoA than RUN, ODD and HIG for the distal tibia
	ODD (60)		23.5±5.1		9.9±4.0	7.8±3.1			SWI showed lower total CSA than HIG for the distal tibia
	POW (17)		27.5±6.3		8.0±4.7	9.1±2.7			SWI showed lower PSM than HIG and ODD for the distal tibia
	RUN (18)		28.9±5.6		12.4±6.7	10.9±3.4			No differences between SWI and CG for the tibial shaft
	CG(50)		24.1±3.4			2.9±1.5			SWI showed lower BMC than POW, ODD and HIG for the tibial shaft
									SWI showed lower CoA, PSM and total CSA than RUN, ODD and HIG for the tibial shaft

ACT =  Average cortical thickness; BMD =  Bone mineral density; CA =  Compression; CG =  Control Group; CoA =  Cortical area; CRI =  Cricketers; CSA =  Cross-sectional area; F =  Female; HIG =  High impact exercises; Imax =  Bending deformation in the maximum plane; Imin =  Bending deformation in the minimum principle plane; JUM =  Jumpers; M =  Men; ODD =  Odd-impact exercises; PMI =  Polar moment of inertia; POW =  Power lifting; PSM =  Polar Section Modulus; RAC =  Racket sports; RUN =  Endurance running; SSI =  Strength strain index; SWI =  Swimmers; TA =  Total bone area; TD =  Torsional deformation; vBMD =  Volumetric bone mineral density; W =  Women.

**Table 3:** Studies using ultrasound.

Study	Participants			Study design	Training years	Training hours/week	Data source	Mesured areas	Outcome
	Subjects	Sex	Age						
Taaffe et al. [41] 2001	SWI & WPO (10)	M	25.5±5.6	Case- control	Began at 14	12	Ultrasound SPA	Calcaneus	Ultrasound attenuation determined by QUS was not significantly different among groups
	JUM (10)		24.9±3.9		Began at 14	12			
	CG (10)		27.7±77.3						
Falk et al. [89] 2003	SWI (21)	F	11.0±0.9	Case- control	>1.5	7.8±3.4	Ultrasound	Radius Tibia	SWI showed lower mean radial SOS values than GYM
	GYM (25)		10.0±0.7		>1.5	4.2±1.7			SWI showed higher tibial SOS values than CG
	CG (21)		10.1±1.1			Physical activite twice per week	Calcium intake		No differences were observed between groups in calcium intake.
Falk et al. [88] 2004	SWI (61)	F	15.9±4.9	Case- control	>1.5	SWI trained 2 to 6 times per week	Ultrasound	Radius Tibia	SWI and CG had similar radial SOS
	CG (71)		15.0±4.0			CG ≤2 times per week			SWI had enhanced tibial SOS values compared to CG
							Calcium intake		No differences were observed between groups in calcium intake, being far below the recommende daily intake in almost all subjects
Yung et al. [90] 2005	SWI (15)	M	20.9±1.3	Case- control	≥2	≥4	Ultrasound	Calcaneus	All QUS parameters were higher in exercise groups compared with the control group
	SOC (15)		21.2±1.7		≥2	≥4			SWI showed lower BUA, and stiffness index scores than SOC and DAN
	DAN (10)		20.6±0.7		≥2	≥4	Calcium intake		No differences in calcium intake among groups
	CG (15)		21.3±1.2						
Falk et al. [86] 2007	SWI & WPO (89)	M	8–23	Case- control	>1.5	4.5–22	Ultrasound	Radius Tibia	Radial SOS measures did not differ between athletes and nonathletic controls
	SOC (97)		8–23		>1.5	4.5–22			SWI showed higher tibial SOS values than CG
	CG (80)								No differences were observed between the SWI and SOC in any of the age groups
							Calcium intake		Reported calcium intake was low in all groups
Velez et al. [35] 2008	SWI (43)	M-F	72.6±6.8	Case- control	–	–	Ultrasound	Calcaneus	SWI showed lower calcaneal stiffnes index than RUN
	RUN (44)		73.3±7.1						No differences in stiffness index between SWI and CG
	CG (87)		75.3±5.4						
Ludwa et al. [87] 2010	SYS (20)	F	15.3±1.2	Case- control	–	SYS≥6	Ultrasound	Radius Tibia	No significant differences between SWI and CG in radial and tibial SOS values
	CG (20)		15.2±1.1				Bone markers		No differences in OC or NTx
									SYS had lower IGF-I concentrations than CG
							Calcium intake		No differences in daily calcium intake among groups although both were below the recommended daily intake.
Shenoy et al. [91] 2012	SWI (40)	M-F	22.4±1.7	Case- control	9.1±3	–	Ultrasound	Radius Tibia	SWI showed lower mean dominant radial, and both dominant and non-dominant tibial SOS than SOF.
	SOF(40)		21.9±1.7		8.3±3				SWI showed higher dominant tibial SOS than the CG
	CG (40)		22.5±1.9						
Czeczuk et al. [49]	SWI1 (11)	F	52.1±3.3	12 month follow-up	Currently not swimming	Current PA 4.8	Ultrasound Calcium intake	Calcaneus LSP	SWI1 showed higher stiffness index values than SWI2
	SWI2 (7)		63.3±4.3			Current PA 6.3			SWI1 and CG1 showed a decrease in the stiffness index values after a 1 year period
	CG1 (11)		50.7±2.2			Current PA 1.4			SWI2 increased by 0.1% their stiffness index values while CG2 decreased 2.4%
	CG2 (7)		60.6±2.3			Current PA 0.6			

BMC =  Bone mineral content; BMD =  Bone mineral density; BUA =  Broadband ultrasound attenuation; CG =  Control group; DAN =  Dancers; DXA =  Dual-energy x-ray absorptiometry; CG =  Control group; GYM =  Gymnasts; JUM =  Jumpers; IGF-I =  Insulin-like growth factor 1; M =  Men; NTx =  Cross-linked N-telopeptide of type I collagen; OC =  Osteocalcin; QUS =  Quantitative ultrasound; RUN =  Runners; SWI =  Swimmers; SWI1 =  Post-menopausal swimmers for less than 5 years ; SWI2 =  Post-menopausal swimmers for more than 5 years; SOC =  Soccer; SOF =  Softball; SOS =  Speed of sound; SYS =  Synchronized swimmers; W =  Women; WPO =  Water polo.

**Table 4:** Studies using MRTA.

Study	Participants			Study design	Training years	Training hours/week	Data source	Measured areas	Outcome
	Subjects	Sex	Age						
Liang et al. [81] 2005	SYS (13)	F	21±0.5	Case- control	10.4±0.5	36	MRTA	Tibia Ulna	EI from the ulna and tibia in each group of athletes was greater than in the CG.
	GYM (8)		20±0.4		13.9±0.6	20			SYS showed lower wrist BMD than GYM and CG
	CG (16)		22±0.1						

EI =  Bone bending Stiffness; CG =  Control group; GYM =  Gymnasts; MRTA =  Mechanical response tissue analyzer; SYS =  Synchronized swimmers.

Regarding the quality assessment; cross-sectional studies (Table S2) were mostly graded with a 4/7 (47 studies), fewer scored 5/7 (7 studies), and only 3 studies were graded with a 6/7. Longitudinal studies (Table S3) were poorly graded with a maximum of 15/33. This was in line with the results obtained by Tooth et al. [20] who designed the checklist and found a mean of 17/33 in the studies that they included in their review [20].

### 1. Bone assessment methods

#### 1.1 BMD and BMC analyzed by photon absorptiometry

The majority of the studies included in this review used photon absorptiometry to assess bone mass in swimmers; in fact 53 of the 64 studies included used this method to evaluate bone mass (Table 1). Dual energy X-ray absorptiometry (DXA) is the most common photon absorptiometry method used. DXA is a two dimensional measure highly influenced by body size [21]. It therefore seems necessary to adjust by covariates to minimize the differences among participants when these are compared. The decision regarding which covariates better adjust the bone mass values is taken by each researcher, taking into account participant age-range, comparison group and so on. For the purposes of this review the final results and authors conclusions presented in each published work were used, regardless of the covariates employed and whether results had been adjusted.

Nilsson et al. [22] first evaluated bone mass in swimmers, other athletes and in a non-athletic control group (CG) aged 18 to 22. They observed higher BMD in the femur of all the athletes than in the CG; however, swimmers did not differ in BMD values when compared with the CG. These findings of similar BMD values in swimmers and CG were reinforced by subsequent studies that also compared swimmers bone mass with CG who performed less than 3 hours of physical activity per week in both male and female subjects, in children [23]–[27], adolescents [28]–[34] young adults [35]–[41] or elderly populations [35]. Some of these studies included a comparison sport group that also showed no differences in BMD when compared with the swimmers [22], [31], [38], [39]. Furthermore, some of the first studies performed on swimmers such as Jacobson et al. [42] or Orwoll et al. [43] as well as other studies found higher arm BMD [16], [42]–[44] or BMC [45], [46] in swimmers than in CG. However, other measured sites in these studies such as lumbar spine [16], [42], [45], a weight bearing zone, or whole body [44], were similar in swimmers and CG. These exclusive higher arm BMD values in swimmers may be due both to the level of force applied by the forearm muscles while swimming, and to the fact that this part of the body is not overly used in daily life by the general population. In fact, Orwoll et al. [43] who included both men and women in their study, found differences only in the male group. According to the authors this was due to the greater forces applied by males, not reached by females who presented values similar to their peer CG. Out of the 50 studies included in this review using DXA, only 2 [45], [47] showed higher whole body BMD in swimmers than in CG. In both studies lean mass, which is well known to influence BMD, [48] was significantly higher in swimmers than in the CG, and was not included as a covariable in the comparisons. This fact may mask some important real differences. Nevertheless, neither of the two cross-sectional studies were graded with a 6/7 in the quality assessment.

Of the 6 studies [16], [42]–[46] that showed higher upper limb BMD values in swimmers than CG, four were performed on adults over 40 years old suggesting that when practiced in the postmenopausal period swimming might reduce the rate of normal bone mass loss accompanying age [42], [43]. However, those studies showing higher upper limb BMD in swimmers, all graded with a 4/7, and others performed with older aged populations [35], did not take into account other physical activities or sedentary behaviours during life, calcium intake [42], [45] or lean mass [42], [43], [45], all of them variables affecting bone. In fact, when Dook et al. [44] controlled by lean mass, the differences in BMD between swimmers and CG disappeared. This may imply that swimming benefits muscle mantainance but the direct effect of swimming on bone mass at these ages is not clear.

Only 2 studies [37], [45] took past physical activity into account: Andreoli et al. [45] performed a retrospective study concluding that physical activity during youth appeared to have a beneficial effect on bone mass later in life. This conclusion underlines the importance of registering past physical activity in studies evaluating bone mass in the elderly population. Greenway et al. [37] did in fact evaluate past, recent and current physical activity in addition to swim participation, and showed that swimmers, who presented fewer cases of lower bone mass than CG, had performed greater amounts of physical activity (excluding swimming) at the ages ranging from 10 to 19. These higher levels of physical activity registered in the study performed by Greenway et al. [37] may be similar physical activity patterns to those of older adult swimmers evaluated in other studies, who showed higher BMD values than CG but whose past physical activity was not registered.

The only longitudinal study performed in postmenopausal former swimmers showed lower BMD and BMC reductions during a one year follow-up in the former swimmers than the sedentary controls [49]. However, these former swimmers performed 3 times more current physical activity than the controls. Physical activity other than swimming may be the cause of higher BMD and should therefore be taken into account in further studies focused on evaluating bone in a later adulthood population.

It would therefore appear that swimming may be beneficial in later adulthood. In spite of these results, out of the 7 aboved mentioned studies [35], [37], [42]–[45], [49] which compared older swimmers with CG, four [35], [42], [44], [45] also included a sport group (SG), showing lower BMD values in swimmers than in the SG, in leg [44], [45], lumbar spine [42] and hip [35].

Lower BMD values in swimmers than other SG, were not exclusive to later adulthood. Many studies also showed lower leg BMD values in adolescent [14], [15], [17], [29], [30] and adult [36], [40], [41], [50]–[53] swimmers. More important than the leg bone mass values are the lumbar spine and hip values where osteoporotic fractures could take place later in life. Focusing on the lumbar spine, lower BMD at this site was also found in children [23], [25], [26], adolescent [17], [29], [47] and adult [36], [54] swimmers when compared to SG. Many studies also found lower values in pelvic bones such as the femoral neck, the femur intertrocanter area or the hip *per se* in children [24]–[26], adolescent [17], [30], [33], [55], [56] and adult [36], [50], [57]–[61] swimmers when compared to gymnasts [17], [24], [25], [36], [55], [56], [60]–[62], track runners [30], [50], [55]–[57], volleyball [36], [50], [57], [58], soccer [33], [57]–[59] or basketball [50], [58], [59] players.

These lower BMD or BMC values in swimmers compared to SG were accompanied in a small, but still relevant number of studies [32], [46], [51], [54], [56], [61] with lower values in swimmers when compared to CG.

Of the 50 studies that measured BMD or BMC only Courteix et al. [24] showed higher values in swimmers than in SG (gymnasts) although only in the skull, while swimmers showed lower BMD values in most of the studied zones. However, head BMD and BMC were higher in swimmers than in gymnasts suggesting that in prepubertal children the increased BMD induced by impact training in the stressed sites of the body could be related to a decreased skull bone mass. This is the only study that showed differences of skull mass and future studies should be performed to confirm this data. Moreover, these differences among groups could be due to bias selection.

In addition to the cross-sectional studies that revealed lower BMD or BMC in swimmers than gymnasts [17], [23]–[26], [36], [44], [53], [60], [61], Taffe et al. [60] performed a longitudinal study also showing lower BMD in swimmers than in gymnasts, but more importantly, showing that during a 12-month follow-up, adult swimmers gained less lumbar spine and femoral neck BMD than their gymnast counterparts. This occurred despite lower initial BMD values in swimmers than gymnasts and was independent of reproductive hormone status. Similar results were found in other adult cohorts [50], [59], and more recently in adolescent swimmers compared to soccer players [15].

This is extremely important because sporting participation, specially during growth, seems to be effective in reducing the prevalence of osteoporosis-related fractures [63]. However, swimmers present similar or lower BMD values compared to CG and lower than their SG and therefore, at most may present osteoporosis values equal to the general population later in life reaching in the European Union an estimated 3.79 million osteoporotic fractures in the year 2000, with an associated estimated cost of 32 billion Euros [64].

To summarize, it seems that swimming does not produce enough power to stimulate bone growth above the regular pattern, with most studies showing similar BMD or BMC values to CG [16], [17], [23], [25], [26], [30], [31], [33]–[36], [40], [43], [44], [57]–[59], [61], [65]–[67]. A deleterious effect of swimming on bone mass has even been demonstrated in some studies, due to the elevated number of hours spent training in a hypogravity environment and therefore avoiding daily impacts [32], [46], [51], [54], [56], [61], [68]. It does also seem that swimming practiced in adulthood or elderly stages of life, may reduce the rate of normal bone mass loss accompanying age [42], [43], [45], although it is unclear if this is a real direct effect of swimming or it is due to a more active lifestyle. Quality assessment sugests a medium quality level of the literature on this subject. Longitudinal studies and well controlled case-control studies including past physical activity history are needed to elucidate the independent effect of swimming in bone development and evolution from childhood to late adulthood.

#### 1.2 Bone geometry and structure

Bone strength is determined by BMD and bone geometric properties [69]–[71]. However, despite the fact that the use of pQCT allows volumetric (vBMD) to be measured, distinguishes between different bone sections and their respective BMD, and is independent of physical size [72], to our knowledge only 4 studies have used this technique in swimmers [73]–[76] (Table 2). To ascertain whether the mechanical properties of bone in response to long-term physical exercise are related to geometric adaptation and not to vBMD in swimmers as they are in jumpers and tennis players [74], [77], [78] would seem to be an important area of study. In order to determine this, bone could be evaluated with pQCT or using DXA combined with other techniques such as magnetic resonance imaging (MRI) [31], [79] or hip structural analysis (HSA) [14]–[17], [67].

A basic means of studying bone structure is by performing a radiography as Xia Qu Ma [80] did in 1992. He performed a radiograph of the anterior side of both humeri and measured length, cortical thickness and diameters at the proximal, middle and distal thirds finding that swimmers had the lowest medial and lateral cortex at both humeri of all the sport groups compared, and that they presented similar values to the CG. Further studies also measured swimmers humeri [73], [74], tibia [74]–[76] and femur [79] with different devices revealing similar [16], [76], [79] or higher cortical cross-sectional areas (CSA) and bone strength indexes [14], [73], [75] in swimmers than in CG. The 3 studies using pQCT that found an improved structure in swimmers than CG were all performed in the same sample age groups, young adults (20 years old). When HSA was used to evaluate bone structure, adolescent [16], [17] or adult [67] (18–35 years old) swimmers presented values similar to CG. These different results could be due to different age samples or to differences between imaging techniques. However, when compared with other sports, swimmers had lower cortical thickness [17], [74], [75], cortical CSA [17], [31], [75], [76] and lower strength indexes [31], [67], [75], [76]. Results were similar independently of the technique used; pQCT [73]–[76], MRI [31] or HSA [14], [17], [67]. The lower cortical mass described in some studies allows a larger medullary cavity CSA in swimmers, resulting in lower trabecular vBMD as found by Nikander et al. [74] and Duncan et al. [31], and a bone with its mass distributed relatively distally from the centroid.

Similar results were found when bone geometry was assesed by Liang et al. [81], using a totally different technique: the mechanical response tissue analyzer (MRTA), invented for the National Aeronautics and Space Administration (NASA) to evaluate bone strength in astronauts after space flight, using the response of a long bone to a low-frequency vibratory stimulus. Results showed that although synchronized swimmers exhibited lower BMD (calculated with a single photon densitometer) wrist values than GC and SG, the bone bending stiffness in the tibia and ulna were greater in synchronized swimmers than in the CG, reinforcing the fact that structure has a great influence on bone strength independently of BMD.

Ferry et al. [15] compared bone geometry during an 8-month period in swimmers and soccer players finding that swimmers did not increase sub-periosteal width, while soccer players did, as well as bone strength indexes such as the cross-sectional moment of inertia and section modulus Z-scores. Buckling ratio (BR) which is an index of bone instability (BR =  maximum distance from the center of mass to the medial or lateral surface divided by the cortical thickness), was the only value improved in swimmers and maintained in soccer players. BR still remained higher in swimmers after the longitudinal period suggesting that swimmers had weaker bone.

In summary, although swimmers may show weaker bone when compared to other weight-bearing sports as a result of smaller cross-sectional areas, the characteristics of the sport may adapt the bone to a higher trabecular CSA [31] and similar [16], [17], [67], [76], [79] or higher bone strength indexes [73]–[75], [81] when compared to a sedentary group, making it more resistant to bending and torsion than a sedentary bone, even though it may have lower BMD. However, studies including both methods of assessment, DXA with HSA and pQCT or MRI, and different sample age groups should be performed in order to test this hypothesis.

#### 1.3 Stiffness, speed of sound and broadband ultrasound attenuation

The use of quantitative ultrasound (QUS) is an option that avoids results biased by differences in body size among subjects. The values of SOS, BUA, and the Stiffness index (SI) derived from both of the former, provided by the QUS, are related to bone density and structure [82] but not to cortical thickness [83]. SI is the default parameter used by the manufacturer for demographic comparison of patient data because SOS and BUA are given as absolute values with no normative values for them. Thus T-scores for these two parameters cannot be calculated [84], [85].

Taaffe et al. [41] compared swimmers with jumpers and a CG observing no differences among the 3 groups in BUA. Similar values between swimmers and other SG were found in only one other study [86]. However, a lack of differences between swimmers and CG was also found in two further studies [35], [87]. In contrast, Falk et al. [86], [88], [89] observed higher tibial SOS values in both male and female swimmers than in the CG in their 3 studies; as others [90], [91] did. Despite the above findings, some of these studies included a SG and with further studies showed that either children [89], adults [90], [91] or elderly [35] swimmers presented lower values than their high impact comparison groups. Showing in 2 of the previous studies lower SI values in swimmers than SG [35], [90].

To our knowledge only one longitudinal 12 month follow-up study using QUS has been performed in swimmers [49]. The study sample was composed of former postmenopausal swimmers that were not swimming when the study took place. Swimmers showed a lower decrease in SI values than CG, although they performed more current physical activity (other than swimming). Therefore it is not possible to confirm that the lower SI decrease was due to swimming (in earlier stages of life) or was a consequence of current physical activity.

We can briefly conclude that swimmers have higher QUS values than CG although these values seem to be higher in high impact sports than in swimmers. It is worth noting that the higher QUS values in swimmers when compared to CG were generally present in the lower limbs. However, the major forces applied in some swimming styles such as crawl or backstroke are applied with the upper limbs. This would suggest that the higher QUS values presented by the swimmers but not by the synchronized swimmers [87], might be due to the push that swimmers perform against the wall, although future studies should be performed comparing swimmers with similar training and history habits who train in a 25 and 50 meter pool to compare their lower limb QUS values.

#### 1.4 Bone markers

Bone is a metabolically active tissue that is constantly changing, with BMD being the result of bone formation and resorption which are closely linked in time and space within the bone multicellular unit [92]. To assess these changes we can measure bone turnover markers which are usually able to provide an early indication of an effect on bone and can be quite sensitive, their main limitations being poor specificity of response and lack of validated connection with the functional outcome [93].

Regarding bone formation markers, several studies showed higher bone-specific alkaline phosphatase (B-ALP) values in swimmers than in CG [47] and SG [40], [47], [94]. However, Morgan et al. [59] found lower values of B-ALP in swimmers than in high impact sports. These diverse results may be due to the fact that the sample ages of the studies were adolescents [47], 20 year old adults [59], [94] and 30 year old adults [40]; all stages of life with different bone dynamics. Another bone formation marker used is osteocalcin (OC), that was lower [57], similar [16], [17], [28], [59], [87] or higher [40] in swimmers than in CG and lower [57] or similar [17], [40], [59] when compared to SG. These differences between studies are a clear example of the difficulties inherent in the use of biochemical markers owing to the wide range of interactions possible depending on age, gender, nutritional status, season and time following intense training [95], [96]. Added to these difficulties and as described by PASSCLAIM [93], the OC molecule exhibits considerable immunological heterogeneity. This, combined with the fact that no internationally recognized assay standard exists, make OC measurements difficult to interpret meaningfully. This may be one of the explanations of the heterogeneity of this bone marker in swimmers.

The use of two bone markers to evaluate bone formation [17], [40], [59] permits differing results to be compared and is therefore a recommended methodology to follow for future studies.

Regarding bone resorption, Cross-linked N-telopeptides of type I collagen (NTx) was the most used biomarker [57], [59], [87], showing no differences between swimmers, CG and other SG. Other resorption markers used were pyridinoline and deoxypiridinoline both lower in male swimmers than judo athletes [94] and higher than CG [47]. Type I collagen C-telopeptide (CTx), another resorption marker that has been recommended by The International Osteoporosis Foundation and the International Federation of Clinical Chemistry [1] was similar [16], [17] or higher in swimmers when compared to CG [40]. This latter point, added to the previously named higher B-ALP values, suggests a higher bone turnover in swimmers than in controls which reflects an intense remodelling process without producing any differences in BMD. Perhaps the use of other techniques to assess bone quality or structure such as pQCT might have shown differences among bones and therefore explained the differences revealed among bone markers.

The Osteoprotegerin(OPG)/Rank-ligand (RANKL) system which is known to have considerable influence on bone formation and degradation, has only been evaluated in one study [17], that showed lower levels of RANKL in swimmers than in gymnasts and controls. Further studies evaluating OPG and RANKL in swimmers are needed in order to confirm these results and elucidate the effect that swimming might have on this system.

Summarizing all the previous studies that include swimmers and bone metabolic markers we can conclude that swimming seems to involve a high bone turnover [40], [47], [94] although in most cases this is not translated into higher BMD. Out of the 14 studies included in this review including bone metabolic markers, only one [87] was performed with a technique other than DXA; QUS, which showed no differences between QUS parameters or bone metabolic markers between swimmers and CG, suggesting that bone metabolic markers might have a high relation to bone structure. Moreover, Maïmoun et al. have performed HSA in two recent studies [16], [17] finding no differences in bone markers nor bone structure between swimmers and CG, thus reinforcing the previous hypothesis. However, further studies including bone metabolic markers and techniques which allow the evaluation of bone structure are needed in order to confirm this hypothesis.

### 2. Factors affecting bone mass

#### 2.1 Hormonal profile

It is well known that hormone concentrations change during growth and vary during or after the practice of exercise[97], [98]. In addition, some parameters of the hormonal profile affect bone metabolism; for example estrogens have been demonstrated to stimulate the proliferation of osteoblasts [99] and insulin-like growth factor-I (IGF-I) has been shown to activate bone turnover [100]. It is therefore important to describe whether hormone concentrations were similar in swimmers and CG or SG.

Lima et al. [47] first found lower testosterone (TT) values in swimmers than in high impact athletes. However, when compared to other non-impact athletes such as cyclists, swimmers showed higher values of TT. Comparison with CG exhibit inconclusive results showing lower [27], and similar [16], [39] values in swimmers. These differences could be due to different training loads as previously suggested by others [101], or due to the different age-range in the samples of the studies [27], [39].

Estradiol levels were also studied, with no differences found between young swimmers, CG and other sports [39]. A similar case is the Luteinizing hormone that was also measured in three studies and also revealed no differences in values between swimmers, CG and other sports in adolescents [16], [47] and adults [39].

Jürimae et al. focused on the influence of Ghrelin (GR) on BMD, showing that GR appeared to be an important hormonal predictor for BMD in swimmers [27]. However, in a further study they showed that GR was not related to measured BMD values in swimmers, suggesting that GR concentration did not have a direct influence on bone mineralization in female swimmers [102].

Jürimae et al. [27], [102], also studied leptin which is directly related to fat mass as well as to BMD in children and adolescents [103], [104] finding similar values in swimmers, GC and sport reference values. Further studies [56] also found similar values in swimmers suggesting that leptin concentration in swimmers is not related to bone mineral parameters.

IGF-I which stimulates endochondral bone formation and activates bone turnover [100] was lower in swimmers than in CG [87] and lower increases during pubertal development were found in a further longitudinal study [27]. However in the latest published study regarding IGF-I, swimmers and CG presented similar values [16]

We can therefore conclude that the values of the majority of the hormones studied were similar in swimmers and CG. However, the concentrations of some of these hormones may vary according to training loads, making the measuring period critical regarding hormone concentrations and associated effects.

#### 2.2 Calcium intake

Most of the studies that registered calcium intake showed no differences among groups. Eigth studies showed significantly higher calcium intake values in swimmers than in CG [14], [15], [32], [34], [35], [40], [49], [105]. Calcium is closely related with bone mass and its intake through diet is crucial. According to the recommended dietary allowance guidelines [106] the studies performed on children [23], [25], [66], [89], adolescents [14], [15], [31], [33], [34], [47], [86], [88], young adults [86], [88], [90] and older adults [37], [44], [49] indicate that swimmers did not reach the recommendations established by these guidelines for calcium intake. However, some of these studies showed higher BMD than reference values independently of calcium intake.

Research has demonstrated that calcium supplementation increases bone mass significantly during childhood and adolescence [107], [108]. A bone-exercise-nutrition interaction exists as shown in previous studies [109]. It would therefore be interesting to perform specific studies evaluating the effect of calcium intake in swimmers in order to ascertain the possible interactions between physical activity, calcium and bone. Other nutritional aspects like magnesium, phosphorus or vitamin D that have not been registered and also affect bone may also be important regarding results. We would therefore suggest that future studies evaluating bone take these variables into account.

#### 2.3 Gender

Gender differences in peak bone mass acquisition are well documented in humans [110], it is therefore important to describe whether swimming might affect these differences.

It seems that results in studies with either male or female participants are similar. Some studies included both genders showing no differences between groups [32], [94]. However, Orwoll et al. [43], who first included both male and female swimmers, and a further study [28] observed higher BMD values in male swimmers than in CG, whereas female swimmers showed similar bone values to the CG. As previously commented, these exclusive differences for the male group were thought to be due to the greater forces generated by males resulting in a greater effect on bone remodelling. On the contrary, Grimston et al. [55] found lower bone mass values only in male swimmers when compared to CG. Different values between genders were also found by Liu et al [75] that showed higher periosteal area, polar moment of inertia and strain strength index only in female swimmers when compared to CG. These higher values only in females could be due to delayed puberty that results in larger periosteal and endocortical area in girls but not in boys [75], female swimmers having the latest menarche in the study and therefore a later puberty. Similar results were found in another study [46] where female swimmers showed higher arm BMD; however, these differences were inexistent between males.

#### 2.4 Age

We have observed that swimming does not seem to negatively affect bone mass and might provide a stronger bone structure than that of the CG, but weaker than that of other impact sports independently of the age group studied: children, adolescents or adults. However, as previously stated, it seems that swimming might be more beneficial later in life. Of the 5 studies performed with DXA that found benefits of swimming compared to CG, 4 were performed in adults over 40 years old, suggesting that swimming may be a beneficial activity to practice in later adulthood in order to maintain bone. It is possible that these higher bone mass values in this older population were due to a more active life than their CG, as found in some studies [49]. Therefore, it would be interesting to take into account in future studies other sports practiced, in addition to swimming, to explain whether the differences within groups might be due to other activities or whether they are exclusive to swimming.

#### 2.5 Training influence

The number of years practiced and weekly hours trained could influence bone in different ways. Unfortunately, this information is not available in all the articles included in this review. Weekly training hours were therefore evaluated when specified by the authors and no evidence of its influence on bone mass was found, in fact, the data presented in each article regarding weekly training hours and years of practice were not clearly specified and therefore cannot be taken into consideration. Training intensity may also differ between groups that perform the same sport modality and train for a similar number of hours. Different intensities can result in different sport performances and also in different body composition and bone adaptations. Magkos et al. [51] were the only researchers that divided swimmers into sprint and endurance swimmers, finding lower BMD values in the latter. The higher BMD values of the sprinters could be due to the higher muscle force and as a result bone stimulation that sprinters perform during training and competition. However, future studies comparing groups that have different training routines should be perfomed to throw more light on this question.

In addition to swimming hours, many swimmers also perform strength training that could also affect bone. This type of training was generally not reported in most of the studies included in this review. However, due to the osteogenic effect that this type of training has, it is important that future studies evaluating bone in swimmers take this parameter into account

### 3. Limitations

The current systematic review excluded non-English and non-Spanish publications; therefore a possible language bias appears. The included studies were too heterogeneous to perform a meta-analysis. The lack of this type of analysis makes it difficult to obtain strong conclusions. Although tables 1, 2, 3 and 4 contain quantitative information on each individual study the classification of the articles according to their bone evaluation method (DXA, pQCT, QUS and MRTA) is not a closed issue.

## Conclusions

Although few studies found lower BMD in swimmers when compared to sedentary controls most of the research shows similar BMD for both groups independently of the age group, except for later adulthood where swimmers presented lower bone mass decrease than sedentary controls, although it is not clear if these differences are sport-specific or are due to the more active life-style reported in swimmers. It would therefore appear that swimming does not negatively affect bone mass. Swimmers mostly showed lower BMD values than any other SG independently of the age of the sample. In fact, longitudinal studies showed lower BMD increases during a season in this specific sport-group. Nevertheless, swimmers showed higher bone turnover values than sedentary controls that were not reflected in higher BMD. This higher bone turnover seems to be associated with a more efficient bone structure of swimmers which appears to be weaker when compared with high impact sports and stronger when compared to sedentary controls, independently of the method of analysis. Many factors may interfere in the effect of swimming on bone, although no differences among groups were found in some of them such as hormone concentrations or calcium intake, the influence as confounders of these factors has not been elucidated.

### Perspective

There are 3 relevant remaining questions:

How many hours per week, years of practice and intensity of training are needed in order to obtain this structure?
Is it possible to obtain this improved structure at any stage of life, or is it only possible during childhood and/or adolescence?
How long does this structure perdure without activity?

Future research is needed to ascertain some of these questions and establish structural benefits of swimming on bone tissue. It is also noteworty that swimming presents lower risks of traumatic fracture than other high impact sports, it is beneficial for cardiovascular fitness [111], and has an important role not only in the promotion of well-being but also in the improvement of muscle strength, which can prevent falls and resulting fractures [112]. Therefore if the previous questions were answered in a positive way, swimming may be a higly benefitial sport to practice regarding bone health.

Future studies in this area should take into consideration the following aspects:

1. Evaluation method should include:
  - Imaging techniques: Two techniques should be employed in each study. DXA is essential and a second method of the researchers choice to evaluate bone structure, preferebly pQCT or MRI.
  - Bone metabolic markers: To evaluate metabolic activity and view bone remodeling process.
2. Physical activity register
  - Accelerometry: In order to evaluate physical activity objectively.
  - Questionnaires: To evaluate past and current physical activity. This might be of extreme importance in studies performed in adults or elderly population.
3. Diet
  To evaluate calcium, phosphorus and vitamin D intake in addition to total energy intake and other nutrients that might affect bone and body composition is of crucial importance.

1. Other confounders
  - Type of swimming: It is important to distinguish, if possible, between sprint swimmers and long distance swimmers due to the fact that they might present different types of training routines and these might involve different types of efforts that might affect bone in a different way.
  - Years of practice, and weekly hours trained: Particularly when performing a study that compares sport disciplines.
  - Complementary weight work: Many swimmers perform weight training in order to improve their performances. This should also be registered and taken into account in future studies.
  - Point of the season: Training loads change according to the season period and this might affect bone that is constantly adapting. When performing studies that compare sports, evaluation should take place in similar load pattern periods of the different sports.
  - Tanner stage: When performing studies with children or adolescents, tanner stage should always be registered as differences in bone might be partly due to maturation status.
  - Menstrual status: This is another important variable to take into account due to the close relation that it has with bone.

## Supporting Information

Table S1 **PRISMA Checklist.**
(DOC)

Table S2 **Quality assessment tool of the cross-sectional included studies.**
(DOCX)

Table S3 **Quality assessment tool of the included longitudinal studies.**
(DOCX)
