# Supplementary material for: Is Bone Tissue Really Affected by Swimming? A Systematic Review
Source: PLoS One. 2013 Aug 7;8(8):e70119. doi: 10.1371/journal.pone.0070119 (PMC3737199; doi:10.1371/journal.pone.0070119)
Supplement: Table S2 — Quality assessment tool of the cross-sectional included studies. (DOCX) [file pone.0070119.s002.docx]

**Table S2. Quality assessment tool of the cross-sectional included studies.**

| **Author and year** | **Appropriate Research Design?** | **Appropriate Recruitment Strategy?** | **Response Rate?** | **Is Sample Representative? (All clinic populations)** | **Objective and Reliable Measures?** | **Power Calculation/ Justification of Numbers?** | **Appropriate Statistical Analysis?** | **Evidence of Bias?** | **Quality Indicators Met** |
| --- | --- | --- | --- | --- | --- | --- | --- | --- | --- |
| Nilsson et al. 1971[22] | Yes | Yes | Not reported | Unclear | Yes | No | Yes | No | 4/7 |
| Jacobson et al. 1984[42] | Yes | Yes | Not reported | Unclear | Yes | No | Yes | No | 4/7 |
| Orwoll et al 1989[43] | Yes | Yes | Not reported | Unclear | Yes | No | Yes | No | 4/7 |
| Heinrich et al.1990[113] | Yes | Yes | Not reported | No | Yes | No | Yes | No | 4/7 |
| Risser et al.1990[54] | Yes | Yes | Not reported | NCAA Division 1 athletes | Yes | No | Yes | Authors state a small sample size | 4/7 |
| McCulloch et al. 1992[32] | Yes | Yes | Not reported | Unclear | Yes | No | Yes | No | 4/7 |
| Xia Qu MA 1992[80] | Yes | Yes | Not reported | No | Yes | No | Yes | No | 4/7 |
| Grimston et al. 1993[55] | Yes | Yes | Not reported | No | Yes | Yes | Yes | A limitation of the study was the inability to recruit sedentary control children | 5/7 |
| Taaffe et al. 1995[61] | Yes | Yes | Not reported | Unclear | Yes | No | Yes | Authors state that nutrition values were not registered and may have contributed to the observed results | 4/7 |
| Lee et al.  1995[58] | Yes | Yes | Not reported | NCAA Division 1A athletes | Yes | Yes | Yes | Authors state a small sample size | 6/7 |
| Fehling et al.  1995[36] | Yes | Yes | Not reported | Unclear | Yes | No | Yes | No | 4/7 |
| Cassell et al.  1996[23] | Yes | Yes | Not reported | No | Yes | No | Yes | No | 4/7 |
| Dook et al.  1996[44] | Yes | Yes | Not reported | Unclear | Yes | No | Yes | Authors state that the inability to obtain significant differences among groups may be due to a small sample size | 4/7 |
| Matsumoto et al. 1997[94] | Yes | Yes | Not reported | Unclear | Yes | No | Yes | Authors state that they did not evaluate calcium intake and therefore cannot consider de effect of nutrition on bone metabolism | 4/7 |
| Emslader et al. 1998[105] | Yes | Yes | Not reported | No | Yes | No | Yes | No | 4/7 |
| Courteix et al. 1998[62] | Yes | Yes | Not reported | No | Yes | No | Yes | No | 4/7 |
| Courteix et al.  1998[25] | Yes | Yes | Not reported | No | Yes | No | Yes | No | 4/7 |
| Taaffe et al. 1999[65] | Yes | Yes | Not reported | No | Yes | No | Yes | Authors state that due to the small subject number, these results are preliminary and require further investigation | 4/7 |
| Courteix et al. 1999[24] | Yes | Yes | Not reported | No | Yes | No | Yes | Authors state that their apparatus forced them to use two different algorithms for measuring skull and other sites that migth bias their results. | 4/7 |
| Kearny  2000[38] | Yes | Yes | Not reported | No | Yes | No | Yes | No attempt was made to control for other weightbearing activities outside of gymnastics and swimming training competitions. No attempt was made to control for lean body weight nor muscular strength as factors in the determination of BMD | 4/7 |
| Creighton et al. 2001[57] | Yes | Yes | Not reported | Unclear | Yes | No | Yes | Authors state that a possible confound to the findings is self-selection | 4/7 |
| Lima et al.  2001[47] | Yes | Yes | Not reported | No | Yes | Yes | Yes | No | 5/7 |
| Morel et al. 2001[52] | Yes | Yes | Not reported | Representative of the general population | Yes | No | Yes | Athletes who are successful and best adapted. | 5/7 |
| Taffe et al. 2001[41] | Yes | Yes | Not reported | Unclear | Yes | Yes | Yes | Authors stated that the early age starting training age of the jumpers may have contributed to gains observed in bone mineralisation | 5/7 |
| Duncan et al.  2002[30] | Yes | Yes | Not reported | State or national level representatives | Yes | Yes | Yes | No | 6/7 |
| Duncan et al.  2002[31] | Yes | Yes | Not reported | State or national level representatives | Yes | Yes | Yes | Subsample of volunteers from a bigger study. | 6/7 |
| Maimoun et al. 2003[39] | Yes | Yes | Not reported | Unclear | Yes | Cannot tell | Yes | No | 4/7 |
| Liu et al. 2003[75] | Yes | Yes | Not reported | No | Yes | No | Yes | No | 4/7 |
| Falk et al. 2003[89] | Yes | Yes | Not reported | No | Yes | No | Yes | No | 4/7 |
| Maïmoun et al. 2004[40] | Yes | Yes | Not reported | Unclear | Yes | No | Yes | No | 4/7 |
| Falk et al. 2004[88] | Yes | Yes | Not reported | No | Yes | No | Yes | No | 4/7 |
| Liang et al. 2005[81] | Yes | Yes | Not reported | Unclear | Yes | No | Yes | Authors stated that the methodology used to quantify bending stiffness may not have been sensitive enough to detect fine structural differences in the long bones of athletes exposed to different types of training. The study was also limited by group size. | 4/7 |
| Yung et al. 2005[90] | Yes | Yes | Not reported | No | Yes | No | Yes | Authors stated that the major limitation of the cross sectional design was the potential bias of self selection in sampling. | 4/7 |
| Bellew et al.  2006[68] | Yes | Yes | Not reported | Unclear | Yes | Yes | Yes | Differences in BMD noted among groups may not be due to differences in sport-specific skeletal loading but rather due to a genetic predisposition. | 5/7 |
| Nikander et al. 2006[74] | Yes | Yes | Not reported | No | Yes | No | Yes | Authors state that in cross-sectional studies of adult athletes, the possibility of self-selection bias is always a concern that cannot be ignored. | 4/7 |
| Magkos et al. 2007[51] | Yes | Yes | Not reported | No | Yes | Yes | Yes | A note should also be made of the relatively small sample size, which requires that our findings be interpreted cautiously | 5/7 |
| Magkos et al. 2007[46] | Yes | Yes | Not reported | Unclear | Yes | No | Yes | Possible self-selection and favorable genetic endowment  among elite athlete populations | 4/7 |
| Mudd et al.  2007[53] | Yes | Yes | 99/135 | Unclear | Yes | No | Yes | Some limitations of the current study include the use of region-of-interest BMD values from a total-body scan and a lack of dietary information, training and injury histories, and detailed menstrual histories. | 5/7 |
| Nichols et al.  2007[114] | Yes | Yes | Not reported | Unclear | Yes | No | Yes | Authors state absence of a non-athlete control group and the lack of measures of bone geometry, which would have provided  estimates of bone strength. | 4/7 |
| Falk et al. 2007[86] | Yes | Yes | Not reported | Unclear | Yes | No | Yes | No | 4/7 |
| Derman et al.  2008[28] | Yes | Yes | Not reported | No | Yes | No | Yes | Authors state that the data is limited to DXA findings because the focus was on BMD; therefore, they are not able to comment on the value of QUS over DEXA. | 4/7 |
| Velez et al. 2008[35] | Yes | Yes | Not reported | Unclear | Yes | No | Yes | Authors state that they were not able to asses athletes´ training and Information on life long physical activity was limited among all participants | 4/7 |
| Jürimäe et al. 2009[27] | Yes | Yes | Not reported | No | Yes | No | Yes | No | 4/7 |
| Shaw et al. 2009[73] | Yes | Yes | Not reported | Unclear | Yes | No | Yes | No | 4/7 |
| Ludwa et al. 2010[87] | Yes | Yes | Not reported | Unclear | Yes | No | Yes | Authors stated small sample size | 4/7 |
| Gruodytè et al. 2010[56] | Yes | Yes | Not reported | Unclear | Yes | No | Yes | No | 4/7 |
| Nikander et al. 2010[76] | Yes | Yes | Not reported | Unclear | Yes | No | Yes | Authors state that cross-sectional studies are not free from bias. Individuals with genetically strong musculature and skeleton may be more likely to start and athletic career in their youth. | 4/7 |
| Dias Quiterio et al. 2011[29] | Yes | Yes | Not reported | No | Yes | No | Yes | Authors state a small number of athletes in each sport group and in the control group and the fact that  all the bone measurements were derived from a whole-body scan. | 4/7 |
| Silva et al.  2011[33] | Yes | Yes | Not reported | No | Yes | No | Yes | No | 4/7 |
| Długołęcka et al.[34]  2011 | Yes | Yes | Not reported | No | Yes | No | Yes | No | 4/7 |
| Ferry et al.  2011[14] | Yes | Yes | Not reported | Unclear | Yes | No | Yes | No | 4/7 |
| Greenway et al. 2012[37] | Yes | Yes | Not reported | Unclear | Yes | No | Yes | No | 4/7 |
| Andreoli et al. 2012[45] | Yes | Yes | Not reported | No | Yes | No | Yes | Authors state that the limitation of this study is that it is a retrospective study and not a longitudinal one | 4/7 |
| Shweta Shenoy et al. 2012[91] | Yes | Yes | Not reported | No | Yes | No | Yes | No | 4/7 |
| Hind et al.[67] 2012 | Yes | Yes | Not reported | Unclear | Yes | No | Yes | Authors state that the participant groups were not equal, with only 10 participants in the group of swimmers, and small number could limit the generalisability of their findings | 4/7 |
| Maïmoun et al.[17] 2013 | Yes | Yes | Not reported | No | Yes | No | Yes | Authors state that the fact that the study was not longitudinal limits their conclusions | 4/7 |
| Narra et al.[79] 2013 | Yes | Yes | Not reported | Unclear | Yes | No | Yes | Authors state that due to the limited spatial resolution of the magnetic resonance image, their findings need to be interpreted with caution | 4/7 |
