# Supplementary material for: Is Bone Tissue Really Affected by Swimming? A Systematic Review
Source: PLoS One. 2013 Aug 7;8(8):e70119. doi: 10.1371/journal.pone.0070119 (PMC3737199; doi:10.1371/journal.pone.0070119)
Supplement: Table S3 — Quality assessment tool of the included longitudinal studies. (DOCX) [file pone.0070119.s003.docx]

**Table S3.** Quality assessment tool of the included longitudinal studies.

|  | 1 | 2 | 3 | 4 | 5 | 6 | 7 | 8 | 9 | 10 | 11 | 12 | 13 | 14 | 15 | 16 | 17 | 18 | 19 | 20 | 21 | 22 | 23 | 24 | 25 | 26 | 27 | 28 | 29 | 30 | 31 | 32 | 33 | TS |
| --- | --- | --- | --- | --- | --- | --- | --- | --- | --- | --- | --- | --- | --- | --- | --- | --- | --- | --- | --- | --- | --- | --- | --- | --- | --- | --- | --- | --- | --- | --- | --- | --- | --- | --- |
| Taffe et al. 1997[60] | N | N | N | Y | Y | N | N | N | N | N | N | N | N | N | Y | Y | N | Y | Y | Y | N | N | Y | Y | N | N | N | N | N | N | N | Y | Y | 11 |
| Carbuhn et al. 2010[50] | Y | Y | N | Y | Y | Y | N | N | N | N | N | N | N | N | Y | Y | N | Y | N | Y | N | N | Y | Y | N | N | N | N | N | N | N | Y | N | 12 |
| Morgan et al. 2011[59] | Y | Y | N | Y | Y | N | N | N | N | N | N | N | N | N | Y | Y | N | Y | N | Y | N | N | Y | Y | N | N | N | N | N | N | N | Y | N | 11 |
| Ferry et al. 2012[15] | Y | Y | N | Y | N | N | N | N | N | N | N | N | N | N | Y | Y | N | Y | Y | Y | N | N | Y | Y | N | N | N | N | N | N | N | Y | N | 11 |
| Czeczuk et al. 2012 [49] | Y | Y | N | Y | Y | N | N | N | N | N | N | N | N | N | Y | Y | N | Y | N | Y | N | N | Y | Y | N | N | N | N | N | N | N | Y | N | 11 |
| Maïmoun et al. 2013 [16] | Y | Y | Y | Y | Y | N | Y | N | N | N | N | N | N | N | Y | Y | N | Y | Y | Y | Y | N | Y | Y | N | N | N | N | N | N | N | Y | N | 15 |
| Czeczelewski et al.[66] 2013 | Y | Y | N | Y | Y | Y | N | N | N | N | N | N | N | N | Y | Y | N | Y | N | Y | N | N | Y | Y | N | N | N | N | N | N | N | Y | N | 12 |

Y=Yes; N=No;TS=Total Score; Ítems from 1 to 33 in order following the checklist criteria from: “Quality of reporting of observational longitudinal research” [20]
